# Crystal structure of *N*,*N*′-dibenzyl-3,3′-di­meth­oxy­benzidine

**DOI:** 10.1107/S2056989018001688

**Published:** 2018-02-02

**Authors:** Hansu Im, Jineun Kim, Changeun Sim, Tae Ho Kim

**Affiliations:** a Department of Chemistry (BK21 plus) and Research Institute of Natural Sciences, Gyeongsang National University, Jinju 52828, Republic of Korea

**Keywords:** crystal structure, hydrogen bond, one-dimensional ladder, *o*-dianisidine

## Abstract

The title compound, (systematic name: *N*,*N*′-dibenzyl-3,3′-dimeth­oxy-1,1′-biphenyl-4,4′-di­amine), C_28_H_28_N_2_O_2_, was synthesized by the reduction of a Schiff base prepared *via* a condensation reaction between *o*-dianisidine and benzaldehyde under acidic conditions. The mol­ecule lies on a crystallographic inversion centre so that the asymmetric unit contains one half-mol­ecule. The biphenyl moiety compound is essentially planar. Two intra­molecular N—H⋯O hydrogen bonds occur. The dihedral angle between the terminal phenyl and phenyl­ene rings of a benzidine unit is 48.68 (6)°. The methyl­ene C atom of the benzyl group is disordered over two sets of sites, with occupancy ratio 0.779 (18):0.221 (18). In the crystal, mol­ecules are connected by hydrogen bonding between *o*-dianisidine O atoms and H atoms of the terminal benzyl groups, forming a one-dimensional ladder-like structure. In the data from DFT calculations, the central biphenyl showed a twisted conformation.

## Abstract 2

The title compound comprises a central *o*-dianisidine unit and terminal benzyl groups. In the mol­ecule, the O atom are involved in intra­molecular hydrogen bonding. In the crystal, adjacent mol­ecules are linked by C—H⋯O inter­actions, forming a one-dimensional ladder along the *a* axis.

## Chemical context   

Benzidine derivatives have received increasing attention in recent years beacuse of their applications in a wide variety of domains, for instance as building blocks in the construction of functionalized organic/organometallic materials and as sensor materials (Hmadeh *et al.*, 2008 ▸; Satapathi, 2015 ▸; Nagaraja *et al.*, 2017 ▸). The chemical and physical properties of benzidine-based compounds have enabled their use in cell biology as staining reagents (Liu *et al.*, 2004 ▸). Benzidine derivatives are also relevant examples of simple redox systems, which could find applications as OLEDs (Zhang *et al.*, 2004 ▸) or electroactive organic polymeric compounds (D’Eramo *et al.*, 1994 ▸). Recently, we have reported copper(I) coordination polymers based on pyromellitic di­imide derivatives, and shown that photoluminescence emission peaks are shifted depending on the solvent (Kang *et al.*, 2015 ▸). In an extension of previous research, we have synthesized a benzidine derivative as a di­amine inter­mediate, in which a benzidine moiety was used instead of a pyromellitic di­imide spacer unit, and report its crystal structure here.
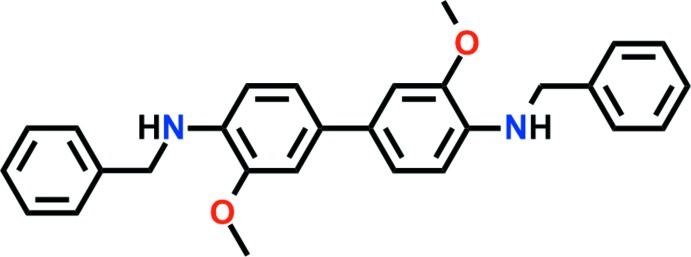


## Structural commentary   

**Figure 1:**
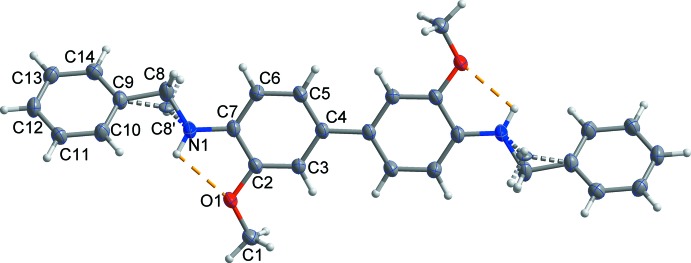
The asymmetric unit of the title compound, with displacement ellipsoids drawn at the 50% probability level. H atoms are shown as small spheres of arbitrary radius and yellow dashed lines represent the intra­molecular N—H⋯O hydrogen bonds. Unlabelled atoms are generated by the symmetry operation (−*x*, −*y*, −*z* + 1).

The mol­ecular structure of the title compound consists of a central di­meth­oxy­benzidine unit and two terminal benzyl groups (Fig. 1 ▸). The mol­ecule lies about a crystallographic inversion centre at the midpoint of the C4—C4(−*x*, −y, −*z* + 1) bond, thus the asymmetric unit contains one half-mol­ecule. The dihedral angle between the terminal phenyl and phenyl­ene rings of a benzidine unit is 48.68 (6)°. Disorder was modelled for the methyl­ene C atom of the benzyl group over two sets of sites with an occupancy ratio of 0.779 (18):0.221 (18). The biphenyl moiety is strictly planar [dihedral angle between rings = 0°; maximum deviation of 0.015 (2) Å for atom C3]. There is no pronounced anisotropy in the aryl anisotropic displacement parameters, indicating that there is no disorder or dynamic twisting process to accommodate the steric crowding of the *ortho* H atoms of the biphenyl moiety (El-Shafei *et al.*, 2003 ▸). The mol­ecular conformation is in part influenced by the formation of weak intra­molecular N1—H1⋯O1 hydrogen bonds that enclose *S*(5) rings (Fig. 1 ▸, Table 1 ▸).

## Supra­molecular features   

**Figure 2:**
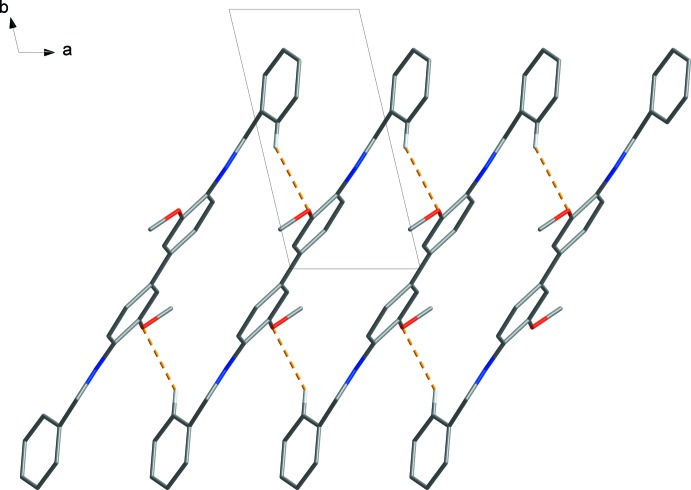
C—H⋯O hydrogen bonds (orange dashed lines) link adjacent mol­ecules. H atoms not involved in inter­molecular inter­actions have been omitted for clarity.

**Figure 3:**
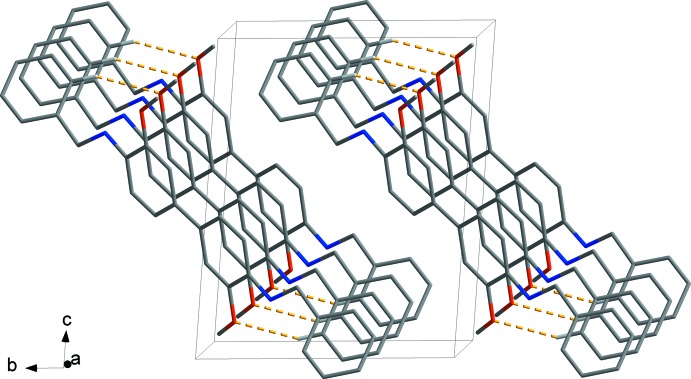
Overall packing diagram of title compound, showing the one-dimensional ladder structure (hydrogen bonds drawn as orange dashed lines). H atoms not involved in inter­molecular inter­actions have been omitted for clarity.

In the crystal, neighbouring mol­ecules are linked by C10—H10⋯O1 hydrogen bonds (Table 1 ▸; yellow dashed lines in Fig. 2 ▸) that generate 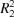
(24) rings. These contacts stack adjacent mol­ecules, forming a one-dimensional ladder-like structure (Fig. 2 ▸). Neighbouring stacks of mol­ecules in these ladders are not connected but lie parallel to the (01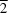) plane (Fig. 3 ▸).

## Database survey   

The Cambridge Database (Version 5.27, last update February 2017; Groom *et al.*, 2016 ▸) reveals polymorphs of related biphenyl derivatives that have both twisted and planar biphenyl conformations (Hoser *et al.*, 2012 ▸). However, in the biphenyl compounds 4,4′-di­amino-2,2′,6,6′-tetra­methyl­biphenyl (Batsanov *et al.*, 2006 ▸), 2,2′-di­chloro-5,5′-dipropoxy­benzidine and 2,2′-dimethyl-5,5′-dipropoxybenzidine (El-Shafei *et al.*, 2004 ▸), in which atoms other than hydrogen are substituted in the *ortho* positions of the biphenyl unit, adopt twisted biphenyl conformations due to steric repulsion between substituted atoms. Hybrid inorganic–organic complexes with benzidine dications display structures with either twisted or planar conformations for the benzidine unit and, in some case, even both conformations (Dobrzycki & Woźniak, 2009 ▸). Related structures with an essentially planar benzidine conformation include 3,3′-dipropoxybenzidine (El-Shafei *et al.*, 2003 ▸), *N*,*N*-bis­(di­phenyl­phosphino)benzidine (Kayan *et al.*, 2012 ▸) and *N,N′*-bis­(4-chloro­benzyl­idene)-3,3′-di­meth­oxy­biphenyl-4,4′-di­amine (Subashini *et al.*, 2011 ▸).

## Theoretical calculations   

**Figure 4:**
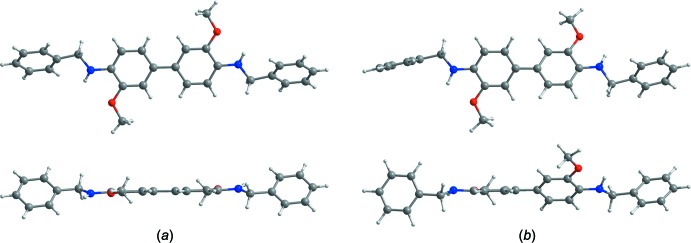
The central biphenyl conformation from the crystallographic data is planar (*a*), while that from the DFT calculations is twisted (*b*).

DFT calculations have been performed to support the experimental values on the basis of the diffraction study using the *GAUSSIAN09* software package (Frisch *et al.*, 2009 ▸). Full geometry optimizations were performed using B3LYP levels of theory with a 6-311G* basis set. The bond lengths of the optimized parameter are in excellent agreement with the experimental crystallographic data (Table 2 ▸). Inter­estingly, however, while the central biphenyl conformation from the crystal structure is found to be planar, that from the DFT calculations shows an angle of 37.67° between the two aromatic rings, Fig. 4 ▸. Furthermore, the dihedral angle between the terminal phenyl and phenyl­ene rings of the title compound is 48.68 (6)° from the crystallographic data but 76.69° from the DFT calculation. Similarly, as a result of the twisted conformation found in the DFT calculations, the lengths of the intra­molecular N—H⋯O hydrogen bonds from the X-ray and DFT calculation data are also slightly different, at 2.33 and 2.21 Å, respectively.

## Synthesis and crystallization   

A mixture of *o*-dianisidine (4.88 g, 20 mmol), benzaldehyde (4.71 g, 40 mmol) and acetic acid (2.47 g, 40 mmol) in 30 mL of toluene and 7 mL of ethanol was heated at refluxed for 6 h. Sodium borohydride (1.62 g, 40 mmol) was added and the mixture was refluxed for two h. After cooling to room temperature, water was added to the reaction mixture. The organic layer was collected and the water layer was extracted with di­chloro­methane. The combined organic layer was dried with anhydrous sodium sulfate then evaporated to give a solid. Column chromatography (silica gel, ethyl acetate/hexane = 30/70 (*v*/*v*) gave the pure product. Crystals suitable for X-ray diffraction analysis were obtained by slow evaporation of an ethyl acetate/*n*-hexane solution (*v*/*v* = 30/70) of the title compound. ^1^H NMR (300 MHz, DMSO): *δ* = 8.31 (*s*, 2H, CHCO), 7.28 (*m*, 10H, phen­yl), 6.64 (*d*, 2H, CCHC), 6.41 (*d*, 2H, CHCN), 5.52 (*t*, 2H, NH), 4.33 (*d*, 4H, CH_2_), 3.88 (*s*, 6H, CH_3_).

## Refinement   

Crystal data, data collection and structure refinement details are summarized in Table 3 ▸. All H atoms were positioned geometrically and refined using a riding model: C—H = 0.95–0.99 Å with *U*_iso_(H) = 1.2*U*_eq_(C). The methyl­ene C8 atom of the benzyl group is disordered over two sets of sites. Their occupancies refined to 0.779 (18) and 0.221 (18).

## Supplementary Material

Crystal structure: contains datablock(s) I, New_Global_Publ_Block. DOI: 10.1107/S2056989018001688/sj5545sup1.cif

Structure factors: contains datablock(s) I. DOI: 10.1107/S2056989018001688/sj5545Isup2.hkl

Supporting information file. DOI: 10.1107/S2056989018001688/sj5545Isup3.cml

CCDC reference: 1820338

Additional supporting information:  crystallographic information; 3D view; checkCIF report

## Figures and Tables

**Table 1:** Hydrogen-bond geometry (Å, °)

*D*—H⋯*A*	*D*—H	H⋯*A*	*D*⋯*A*	*D*—H⋯*A*
C10—H10⋯O1^i^	0.95	2.66	3.400 (2)	135
N1—H1⋯O1	0.88	2.33	2.6464 (19)	101

Symmetry code: (i) 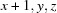.

**Table 2:** Experimental and calculated bond lengths (Å)

Bond	X-ray	B3LYP (6–311G*)
O1—C1	1.425 (2)	1.4208
O1—C2	1.374 (3)	1.3744
N1—C7	1.394 (2)	1.3872
N1—C8	1.438 (5)	1.4567
C2—C3	1.378 (2)	1.3859
C3—C4	1.399 (2)	1.4104
C4—C5	1.389 (2)	1.3951
C5—C6	1.386 (2)	1.3964
C6—C7	1.385 (2)	1.3972
C2—C7	1.408 (2)	1.4189
C8—C9	1.498 (6)	1.5139
C9—C10	1.389 (3)	1.400)
C10—C11	1.379 (3)	1.3921
C11—C12	1.380 (2)	1.3966
C12—C13	1.377 (3)	1.3923
C13—C14	1.383 (3)	1.3965
C9—C14	1.382 (2)	1.3976
C4—C4^i^	1.491 (2)	1.4823

Symmetry code: (i) −*x*, −*y*, −*z* + 1.

**Table 3:** Experimental details

Crystal data	
Chemical formula	C_28_H_28_N_2_O_2_
*M*_r_	424.52
Crystal system, space group	Triclinic, *P* 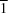
Temperature (K)	173
*a*, *b*, *c* (Å)	4.7089 (2), 9.6760 (4), 12.1952 (5)
α, β, γ (°)	93.387 (3), 92.165 (2), 103.180 (2)
*V* (Å^3^)	539.32 (4)
*Z*	1
Radiation type	Mo *K*α
μ (mm^−1^)	0.08
Crystal size (mm)	0.31 × 0.18 × 0.06

Data collection	
Diffractometer	Bruker APEXII CCD
Absorption correction	Multi-scan (*SADABS*; Bruker, 2014 ▸)
*T*_min_, *T*_max_	0.659, 0.746
No. of measured, independent and observed [*I* > 2σ(*I*)] reflections	6528, 1888, 1683
*R*_int_	0.019
(sin θ/λ)_max_ (Å^−1^)	0.594

Refinement	
*R*[*F* ^2^ > 2σ(*F* ^2^)], *wR*(*F* ^2^), *S*	0.048, 0.145, 1.10
No. of reflections	1888
No. of parameters	156
No. of restraints	6
H-atom treatment	H-atom parameters constrained
Δρ_max_, Δρ_min_ (e Å^−3^)	0.37, −0.60

Computer programs: *APEX2* and *SAINT* (Bruker, 2014 ▸), *SHELXS97* and *SHELXTL* (Sheldrick, 2008 ▸), *SHELXL2014* (Sheldrick, 2015 ▸), *DIAMOND* (Brandenburg, 2010 ▸) and *publCIF* (Westrip, 2010 ▸).

## supplementary crystallographic information

### Crystal data

**Table:**

C_28_H_28_N_2_O_2_	*Z* = 1
*M**_r_* = 424.52	*F*(000) = 226
Triclinic, *P*1	*D*_x_ = 1.307 Mg m^−^^3^
*a* = 4.7089 (2) Å	Mo *K*α radiation, λ = 0.71073 Å
*b* = 9.6760 (4) Å	Cell parameters from 5519 reflections
*c* = 12.1952 (5) Å	θ = 2.6–28.0°
α = 93.387 (3)°	µ = 0.08 mm^−^^1^
β = 92.165 (2)°	*T* = 173 K
γ = 103.180 (2)°	Plate, yellow
*V* = 539.32 (4) Å^3^	0.31 × 0.18 × 0.06 mm

### Data collection

**Table:**

Bruker APEXII CCD diffractometer	1683 reflections with *I* > 2σ(*I*)
φ and ω scans	*R*_int_ = 0.019
Absorption correction: multi-scan (SADABS; Bruker, 2014)	θ_max_ = 25.0°, θ_min_ = 1.7°
*T*_min_ = 0.659, *T*_max_ = 0.746	*h* = −5→5
6528 measured reflections	*k* = −11→11
1888 independent reflections	*l* = −13→14

### Refinement

**Table:**

Refinement on *F*^2^	6 restraints
Least-squares matrix: full	Hydrogen site location: inferred from neighbouring sites
*R*[*F*^2^ > 2σ(*F*^2^)] = 0.048	H-atom parameters constrained
*wR*(*F*^2^) = 0.145	*w* = 1/[σ^2^(*F*_o_^2^) + (0.0797*P*)^2^ + 0.2226*P*] where *P* = (*F*_o_^2^ + 2*F*_c_^2^)/3
*S* = 1.10	(Δ/σ)_max_ < 0.001
1888 reflections	Δρ_max_ = 0.37 e Å^−^^3^
156 parameters	Δρ_min_ = −0.60 e Å^−^^3^

### Special details

**Table:**

Geometry. All esds (except the esd in the dihedral angle between two l.s. planes) are estimated using the full covariance matrix. The cell esds are taken into account individually in the estimation of esds in distances, angles and torsion angles; correlations between esds in cell parameters are only used when they are defined by crystal symmetry. An approximate (isotropic) treatment of cell esds is used for estimating esds involving l.s. planes.

### Fractional atomic coordinates and isotropic or equivalent isotropic displacement parameters (Å^2^)

**Table:**

	*x*	*y*	*z*	*U*_iso_*/*U*_eq_	Occ. (<1)
O1	0.2453 (3)	0.22554 (13)	0.81729 (9)	0.0350 (4)	
N1	0.6872 (3)	0.38961 (16)	0.72486 (12)	0.0367 (4)	
H1	0.6890	0.3862	0.7968	0.044*	
C1	−0.0076 (4)	0.15213 (19)	0.86818 (14)	0.0355 (4)	
H1A	−0.1827	0.1701	0.8312	0.053*	
H1B	0.0024	0.1860	0.9459	0.053*	
H1C	−0.0163	0.0498	0.8625	0.053*	
C2	0.2594 (3)	0.19646 (17)	0.70622 (13)	0.0275 (4)	
C3	0.0682 (3)	0.08840 (16)	0.64431 (13)	0.0270 (4)	
H3	−0.0911	0.0316	0.6786	0.032*	
C4	0.1022 (3)	0.06000 (16)	0.53238 (13)	0.0261 (4)	
C5	0.3347 (4)	0.14802 (18)	0.48559 (14)	0.0328 (4)	
H5	0.3646	0.1318	0.4098	0.039*	
C6	0.5243 (4)	0.25895 (18)	0.54660 (14)	0.0329 (4)	
H6	0.6790	0.3178	0.5114	0.039*	
C7	0.4931 (3)	0.28575 (17)	0.65775 (14)	0.0284 (4)	
C8	0.8843 (16)	0.5024 (3)	0.6758 (5)	0.0414 (13)	0.779 (18)
H8A	0.7710	0.5531	0.6291	0.050*	0.779 (18)
H8B	1.0144	0.4613	0.6282	0.050*	0.779 (18)
C8'	0.791 (3)	0.4999 (12)	0.7085 (11)	0.023 (3)	0.221 (18)
H8'1	0.6326	0.5513	0.7158	0.028*	0.221 (18)
H8'2	0.8268	0.4949	0.6291	0.028*	0.221 (18)
C9	1.0656 (4)	0.60586 (18)	0.76161 (15)	0.0336 (4)	
C10	1.1686 (4)	0.56526 (18)	0.86005 (15)	0.0373 (5)	
H10	1.1067	0.4695	0.8787	0.045*	
C11	1.3596 (4)	0.66246 (19)	0.93095 (14)	0.0368 (4)	
H11	1.4302	0.6334	0.9978	0.044*	
C12	1.4480 (4)	0.80198 (18)	0.90472 (15)	0.0370 (4)	
H12	1.5781	0.8693	0.9538	0.044*	
C13	1.3474 (4)	0.84355 (18)	0.80734 (15)	0.0357 (4)	
H13	1.4098	0.9394	0.7889	0.043*	
C14	1.1558 (4)	0.74595 (18)	0.73633 (14)	0.0339 (4)	
H14	1.0855	0.7754	0.6696	0.041*	

### Atomic displacement parameters (Å^2^)

**Table:**

	*U*^11^	*U*^22^	*U*^33^	*U*^12^	*U*^13^	*U*^23^
O1	0.0359 (7)	0.0396 (7)	0.0222 (6)	−0.0042 (5)	0.0004 (5)	−0.0061 (5)
N1	0.0347 (8)	0.0398 (9)	0.0262 (8)	−0.0081 (7)	0.0009 (6)	−0.0086 (6)
C1	0.0359 (9)	0.0416 (10)	0.0252 (9)	0.0021 (7)	0.0029 (7)	−0.0022 (7)
C2	0.0303 (8)	0.0285 (8)	0.0224 (8)	0.0056 (6)	−0.0033 (6)	−0.0015 (6)
C3	0.0277 (8)	0.0256 (8)	0.0253 (8)	0.0017 (6)	−0.0015 (6)	0.0008 (6)
C4	0.0270 (8)	0.0258 (8)	0.0244 (8)	0.0050 (7)	−0.0047 (7)	−0.0001 (6)
C5	0.0333 (9)	0.0375 (9)	0.0228 (8)	−0.0001 (7)	−0.0019 (7)	−0.0028 (7)
C6	0.0307 (9)	0.0339 (9)	0.0286 (9)	−0.0033 (7)	0.0007 (7)	0.0001 (7)
C7	0.0265 (8)	0.0285 (8)	0.0273 (9)	0.0025 (6)	−0.0043 (6)	−0.0023 (6)
C8	0.048 (2)	0.0322 (14)	0.037 (3)	−0.0026 (15)	−0.014 (2)	−0.0002 (13)
C8'	0.022 (4)	0.029 (4)	0.018 (4)	0.005 (3)	0.011 (3)	0.003 (3)
C9	0.0356 (9)	0.0290 (9)	0.0328 (9)	0.0032 (7)	−0.0054 (7)	−0.0041 (7)
C10	0.0454 (10)	0.0255 (8)	0.0366 (10)	0.0003 (7)	−0.0055 (8)	0.0017 (7)
C11	0.0429 (10)	0.0362 (10)	0.0272 (9)	0.0023 (8)	−0.0065 (8)	0.0008 (7)
C12	0.0346 (9)	0.0331 (9)	0.0367 (10)	−0.0027 (7)	−0.0055 (8)	−0.0056 (7)
C13	0.0351 (9)	0.0276 (9)	0.0405 (10)	−0.0009 (7)	0.0001 (8)	0.0033 (7)
C14	0.0368 (9)	0.0337 (9)	0.0298 (9)	0.0055 (7)	−0.0010 (7)	0.0033 (7)

### Geometric parameters (Å, º)

**Table:**

O1—C2	1.374 (2)	C6—H6	0.9500
O1—C1	1.425 (2)	C8—C9	1.498 (3)
N1—C8'	1.100 (12)	C8—H8A	0.9900
N1—C7	1.394 (2)	C8—H8B	0.9900
N1—C8	1.438 (7)	C8'—C9	1.549 (11)
N1—H1	0.8800	C8'—H8'1	0.9900
C1—H1A	0.9800	C8'—H8'2	0.9900
C1—H1B	0.9800	C9—C14	1.382 (2)
C1—H1C	0.9800	C9—C10	1.389 (3)
C2—C3	1.378 (2)	C10—C11	1.379 (2)
C2—C7	1.408 (2)	C10—H10	0.9500
C3—C4	1.399 (2)	C11—C12	1.379 (2)
C3—H3	0.9500	C11—H11	0.9500
C4—C5	1.389 (2)	C12—C13	1.377 (3)
C4—C4^i^	1.491 (3)	C12—H12	0.9500
C5—C6	1.386 (2)	C13—C14	1.383 (2)
C5—H5	0.9500	C13—H13	0.9500
C6—C7	1.385 (2)	C14—H14	0.9500
			
C2—O1—C1	117.19 (12)	C9—C8—H8A	109.3
C8'—N1—C7	129.4 (6)	N1—C8—H8B	109.3
C7—N1—C8	119.6 (2)	C9—C8—H8B	109.3
C7—N1—H1	120.2	H8A—C8—H8B	108.0
C8—N1—H1	120.2	N1—C8'—C9	131.8 (9)
O1—C1—H1A	109.5	N1—C8'—H8'1	104.3
O1—C1—H1B	109.5	C9—C8'—H8'1	104.3
H1A—C1—H1B	109.5	N1—C8'—H8'2	104.3
O1—C1—H1C	109.5	C9—C8'—H8'2	104.3
H1A—C1—H1C	109.5	H8'1—C8'—H8'2	105.6
H1B—C1—H1C	109.5	C14—C9—C10	118.83 (15)
O1—C2—C3	124.56 (15)	C14—C9—C8	117.8 (2)
O1—C2—C7	114.59 (14)	C10—C9—C8	123.1 (2)
C3—C2—C7	120.84 (15)	C14—C9—C8'	124.5 (4)
C2—C3—C4	121.84 (15)	C10—C9—C8'	114.0 (5)
C2—C3—H3	119.1	C11—C10—C9	120.71 (16)
C4—C3—H3	119.1	C11—C10—H10	119.6
C5—C4—C3	116.84 (15)	C9—C10—H10	119.6
C5—C4—C4^i^	122.18 (18)	C10—C11—C12	119.89 (16)
C3—C4—C4^i^	120.98 (18)	C10—C11—H11	120.1
C6—C5—C4	121.75 (16)	C12—C11—H11	120.1
C6—C5—H5	119.1	C13—C12—C11	119.95 (16)
C4—C5—H5	119.1	C13—C12—H12	120.0
C7—C6—C5	121.41 (16)	C11—C12—H12	120.0
C7—C6—H6	119.3	C12—C13—C14	120.10 (16)
C5—C6—H6	119.3	C12—C13—H13	120.0
C6—C7—N1	124.04 (15)	C14—C13—H13	120.0
C6—C7—C2	117.31 (15)	C9—C14—C13	120.52 (16)
N1—C7—C2	118.54 (15)	C9—C14—H14	119.7
N1—C8—C9	111.4 (4)	C13—C14—H14	119.7
N1—C8—H8A	109.3		
			
C1—O1—C2—C3	−9.3 (2)	C3—C2—C7—N1	−177.38 (14)
C1—O1—C2—C7	171.68 (14)	C7—N1—C8—C9	177.7 (3)
O1—C2—C3—C4	−176.98 (14)	C7—N1—C8'—C9	−160.1 (9)
C7—C2—C3—C4	1.9 (2)	N1—C8—C9—C14	−149.9 (3)
C2—C3—C4—C5	−1.4 (2)	N1—C8—C9—C10	36.3 (6)
C2—C3—C4—C4^i^	178.26 (16)	N1—C8'—C9—C14	−175.1 (12)
C3—C4—C5—C6	−0.1 (3)	N1—C8'—C9—C10	−13.8 (19)
C4^i^—C4—C5—C6	−179.73 (17)	C14—C9—C10—C11	−0.6 (3)
C4—C5—C6—C7	1.0 (3)	C8—C9—C10—C11	173.1 (4)
C5—C6—C7—N1	175.71 (16)	C8'—C9—C10—C11	−163.1 (6)
C5—C6—C7—C2	−0.5 (3)	C9—C10—C11—C12	0.6 (3)
C8'—N1—C7—C6	43.5 (11)	C10—C11—C12—C13	−0.6 (3)
C8—N1—C7—C6	18.3 (4)	C11—C12—C13—C14	0.6 (3)
C8'—N1—C7—C2	−140.3 (11)	C10—C9—C14—C13	0.6 (3)
C8—N1—C7—C2	−165.6 (3)	C8—C9—C14—C13	−173.5 (4)
O1—C2—C7—C6	178.08 (14)	C8'—C9—C14—C13	161.1 (7)
C3—C2—C7—C6	−0.9 (2)	C12—C13—C14—C9	−0.6 (3)
O1—C2—C7—N1	1.6 (2)		

Symmetry code: (i) −*x*, −*y*, −*z*+1.

### Hydrogen-bond geometry (Å, º)

**Table:**

*D*—H···*A*	*D*—H	H···*A*	*D*···*A*	*D*—H···*A*
C10—H10···O1^ii^	0.95	2.66	3.400 (2)	135
N1—H1···O1	0.88	2.33	2.6464 (19)	101

Symmetry code: (ii) *x*+1, *y*, *z*.
